# Grant-Free NOMA: A Low-Complexity Power Control through User Clustering

**DOI:** 10.3390/s23198245

**Published:** 2023-10-04

**Authors:** Abdulkadir Celik

**Affiliations:** Computer, Electrical, and Mathematical Sciences & Engineering (CEMSE) Division, King Abdullah University of Science and Technology (KAUST), Thuwal 23955-6900, Saudi Arabia; abdulkadir.celik@kaust.edu.sa

**Keywords:** machine-type communications, massive connectivity, power domain, grant-free, non-orthogonal multiple access, Internet of Things, resource allocation, user pairing, clustering

## Abstract

Non-orthogonal multiple access (NOMA) has emerged as a promising solution to support multiple devices on the same network resources, improving spectral efficiency and enabling massive connectivity required by ever-increasing Internet of Things devices. However, traditional NOMA schemes operate in a grant-based fashion and require channel-state information and power control, which hinders its implementation for massive machine-type communications. Accordingly, this paper proposes synchronous grant-free NOMA (GF-NOMA) frameworks that effectively integrate user equipment (UE) clustering and low-complexity power control to facilitate the power-reception disparity required by the power-domain NOMA. Although single-level GF-NOMA (SGF-NOMA) designates an identical transmit power for all UEs, multi-level GF-NOMA (MGF-NOMA) groups UEs into partitions based on the sounding reference signals strength and assigns partitions with different identical power levels. Based on the objective of interest (e.g., max–sum or max–min rate), the proposed UE clustering scheme iteratively admits UEs to form clusters whose size is dynamically determined based on the number of UEs and available resource blocks (RBs). Once the UEs are acknowledged with power levels and allocated RBs through random-access response (RAR) messages, UEs can transmit anytime without grant acquisition. Numerical results show that the proposed GF-NOMA frameworks can compute clusters in the order of milliseconds for hundreds of UEs. The MGF-NOMA can reach up to 96–99% of the optimal benchmark max–sum rate, and the SGF-NOMA reaches 87% of the optimal benchmark max–sum rate at the same power consumption. Since the MGF-NOMA and optimal benchmark enforce the strongest and weakest channel UEs to transmit at maximum and minimum transmit powers, respectively, the SGF-NOMA also offers a significantly higher energy consumption fairness and network lifetime as all UEs consume equal transmit powers. Although the MGF-NOMA delivers an inferior max–min rate performance, the SGF-NOMA is shown to reach 3e6 MbpJ energy efficiency compared to the 1e7 MbpJ benchmark.

## 1. Introduction

The ever-increasing demand for connectivity in today’s world is driving continuous evolution in wireless networks. With the advent of the digital age, there is an increasing emphasis on designing network architectures that can seamlessly integrate with various forms of communication and smart technologies. As envisioned by the International Telecommunication Union (ITU), future wireless networks are expected to be versatile enough to cater to the diverse quality of service (QoS) requirements for distinct types of network usage scenarios [1], which can be broadly categorized into three paradigms:

- **Enhanced Mobile Broadband (eMBB):** This primarily focuses on supporting traditional human-type communications (HTC), emphasizing high data rates, broadband services, and providing high-capacity connectivity [2].
- **Massive Machine-Type Communications (mMTC):** As we move towards a more interconnected world, there is a surge in the deployment of the Internet of Things (IoT) devices [3]. mMTC focuses on ensuring that cellular networks can handle the immense connectivity demand from billions of these devices. Unlike eMBB, the emphasis here is not on high data rates but on ensuring reliable, efficient communication for devices that might transmit data sporadically, often in very short packets [4].
- **Ultra-Reliable Low-Latency Communications (URLLC):** In applications like autonomous vehicles or industrial automation, it is crucial to have instantaneous communication with near-zero latency and ultra-high reliability [5]. URLLC has been tailored to meet reliability and latency requirements beyond the perception of a human.

Traditional cellular networks predominantly operated on contention-based channel access methods orthogonally allocate radio resource blocks (RBs) to devices/user equipment (UEs) through the physical random-access channel (PRACH), where the foundational mechanism a four-way handshake [6]: (1) Every device randomly chooses one out of the available preambles and sends it to the BS; (2) the BS sends a random-access response (RAR) message including information about allocated RBs and timing advance for synchronization; (3) following the RAR message reception, devices make a radio resource control (RRC) connection request through the Physical UL shared channel (PUSCH); and (4) the BS sets up RRC connection by sending information of allocated RBs to all devices by specifying their terminal identity. Although device and user equipment are terms typically preferred for MTC and HTC, respectively, this paper uses them interchangeably, as the proposed methods are not limited to the communication type. This handshake ensures not only the establishment of communication between the base station (BS) and the device but also addresses key requirements such as initial access, uplink synchronization, data transmission, acknowledgment responses, and handover management, among other functionalities [7].

However, as efficient as this grant-based (GB) mechanism may seem for eMBB scenarios, where the sheer volume of data might overshadow the signaling overhead, it does show its limitations in the face of mMTC. Given the vast number of IoT devices transmitting short packets intermittently, the delay from this GB process becomes a critical performance bottleneck [8]. Non-orthogonal multiple access (NOMA) is a prospective multiple-access technique envisioned to augment spectral efficiency in wireless communication systems [9]. Unlike traditional orthogonal multiple-access (OMA) methods, where users are distinctly allocated resources to avert interference, NOMA facilitates the simultaneous transmission of multiple users over identical resources, such as time, frequency, and space, yielding various types of NOMA schemes [10]:

Power-domain NOMA (PD-NOMA) primarily relies on superimposing signals from multiple users in the power domain before actual transmission [11], which is made possible by assigning varying power levels to the signals of the different users. The receiver then employs a mechanism known as successive interference cancellation (SIC) to segregate the signals of the individual users [12]. The user signal with the highest power level is decoded first. Subsequently, its influence is removed from the combined signal, allowing the next user’s signal to be decoded. This process is reiterated until all signals are decoded. The allure of PD-NOMA emerges especially in scenarios characterized by heterogeneous user channel conditions, where it often demonstrates enhanced spectral efficiency compared to OMA techniques.

In parallel, code-domain NOMA (CD-NOMA) is another vibrant domain, occasionally referred to by names such as sparse code multiple access (SCMA) [13] or multi-user shared access (MUSA) [14], which hinge on employing distinct codebooks for different users. Essentially, the data of each UE is diffused across several resource elements, each having a specific spreading factor. On the receiving end, techniques leveraging multi-dimensional codebooks or multi-user detection come into play to extricate the overlapping users. The cardinal advantage of this method is its enablement of an overloading factor that allows more users to be served simultaneously than the orthogonal resources available. Such a characteristic provides the system with added flexibility and superior spectral efficiency.

Furthermore, spatial NOMA [15] incorporates the principles of multiple-input multiple-output (MIMO) technology by using the spatial domain, i.e., the multiple antennas at the transmitter and/or receiver, to serve multiple users simultaneously over the same frequency resource. The spatial differences of the users’ channels are exploited to differentiate and decode the overlapping signals. When combined with techniques from PD-NOMA or CD-NOMA, this spatial-based differentiation offers another dimension to improve spectral efficiency and user connectivity. In addition to these primary schemes, hybrid approaches amalgamating features from multiple topologies have also been explored by researchers [16]. Such hybrid strategies aim to assimilate various domains’ strengths, enhancing the robustness and efficiency of wireless communication systems.

PD-NOMA is the most studied and promising approach among these primary NOMA schemes. However, it brings forth its set of challenges, especially concerning the non-convex and combinatorial complexities of power control, user clustering, and the excessive signaling overhead due to the need for channel-state information (CSI) [17,18]. Grant-free NOMA (GF-NOMA) manifests as an evolved facet of the NOMA paradigm, designed meticulously to mitigate certain intrinsic challenges observed in GB-NOMA systems, predominantly in PD-NOMA. In mMTC, many IoT devices frequently exhibit sporadic data transmission patterns and often remain dormant for extended durations, transmitting exclusively upon detecting specific events. The aforementioned GB-NOMA schemes may demonstrably falter in efficiency in such traffic patterns. The consequent overhead spawned by this iterative request-grant protocol, particularly when dealing with concise data packets, emerges as a substantial bottleneck in networks with dense deployments. There are three main advantages of GF-NOMA schemes:

1. **Signaling Overhead Attenuation:** A primary impetus propelling GF-NOMA’s inception is its prowess in truncating the signaling overhead, an inevitable byproduct of recurrent grant access requests in ultra-dense networks [19]. GF-NOMA can streamline network operations, allowing devices to transmit without waiting for prior scheduling, especially for short and sporadic data transmissions.
2. **Power Control Dynamics:** Although classical PD-NOMA is profoundly reliant on power control to delineate users, in the GF-NOMA context, this emphasis is palpably attenuated, albeit not rendered obsolete [20]. Devices might necessitate intermittent power calibrations to ascertain the decodability of their signals at the receiver, especially in the presence of potential interference.
3. **Latency Minimization:** Another salient advantage offered by GF-NOMA is its potential to curtail latency. By obviating the inherent latency in grant-based systems, devices experience expedited data relay, a facet paramount for applications with a penchant for URLLC [21].

In encapsulation, GF-NOMA epitomizes a pivot towards a more structured, adaptable, and efficient wireless communication, especially in network landscapes characterized by density and unpredictability. Although it embodies the promise of aligning with futuristic network aspirations, especially those overcrowded with many IoT devices, GF-NOMA is not devoid of challenges. Accordingly, this paper will focus on providing a fast yet efficient user clustering approach with implicit power control mechanisms, allowing grant-free operation of users.

### 1.1. Related Work

The GF-NOMA can be categorized into synchronous and asynchronous depending on whether it relies on the abovementioned PRACH mechanism. Once UEs perform PRACH and become aligned with the BS in the synchronous GF-NOMA, there is no need for further grant acquisition (GA). On the other hand, the asynchronous GF-NOMA does not need any access or GA processes. Noting that there exist various GF-NOMA schemes exploiting different domains of non-orthogonality (e.g., power, spreading, scrambling, interleaving, etc.) [22], we restrict this section with literature on power-domain GF-NOMA as it is the main focus of the paper. Early GF-NOMA schemes integrated slotted-ALOHA protocols with PD-NOMA [23,24,25], where the BS estimated the number of active devices by various statistical tools (e.g., hypothesis testing) and allow devices independently select predetermined power levels. Stochastic geometry has also been used as an effective tool to analyze the performance of GF-NOMA [26,27,28]. In [26], the authors proposed a semi-GF-NOMA scheme to improve spectral efficiency by multiplexing GB and GF devices on the same RB. By leveraging stochastic geometry techniques, they developed a dynamic protocol that reduces GF devices’ interference to a large extent compared to the open-loop protocol benchmark. This work was further studied in [27] to analyze ergodic rates by deriving the closed-form analytical and approximated expressions. In [28], Liu et al. exploited compressive sensing and stochastic geometry tools to model, analyze, and optimize the GF-NOMA scheme, where active devices were allowed to send preambles and data symbols without a need for GA. In [29], Fayaz et al. proposed an open-loop power control technique, where each UE acts as an agent and power levels are learned through a multi-agent deep Q-network. In [30], Abbas et al. developed a framework treating collisions as interference and deriving approximate expressions of the outage probability and throughput using Poisson point processes and ordered statistics. In [31], Dogan et al. introduced a GF approach for URLLC based on index modulation-based orthogonal frequency division multiplexing (OFDM-IM) to reduce the impact of the collision on the URLLC and achieved 99.999% success probability within 1 ms.

In recent years, there has been a considerable exploration into integrating deep-learning (DL) techniques with GF-NOMA schemes that can substantially improve efficiency and performance, especially for mMTC and URLLC. For instance, Liu et al. [32] introduced a novel learning framework for multiple configured-grants GF-NOMA systems designed to comply with URLLC requirements. They employed a cooperative Multi-Agent Double Deep Q-Network (MA-DDQN) to optimize channel resource allocations, which improved latency and reliability performance, underlining the potential of DL techniques in enhancing URLLC. The use of DL in dynamic resource configuration was further developed in [33], wherein Liu et al. developed an MA-DQN framework for signature-based GF-NOMA, demonstrating a significant improvement in heavy traffic performance for URLLC services. On the other hand, Zhang et al. [34] proposed two efficient Bayesian learning algorithms, which significantly reduced the computational complexity of GF-NOMA systems, showing the benefits of DL in computational efficiency. Their later work [35] further utilized Sparse Bayesian Learning (SBL) approaches to tackle the multi-user detection problem in GF-NOMA and demonstrated the capacity of DL to handle complex detection problems in mMTC.

Moreover, the use of DL has also been extended to address challenges in user activity detection and channel estimation in GF-NOMA. Yu et al. [36] introduced a novel DL architecture, UAD-CE-NN, which showed higher detection accuracy, especially with short preamble sequences. Meanwhile, Khan et al. [37] presented a Deep Neural Network (DNN)-based approach for active user detection in GF-NOMA with sparse spreading. Their active user enumeration and identification method demonstrated substantial performance improvements over traditional methods. Furthermore, Cao et al. have focused on the security aspects of semi-grant-free NOMA transmission [38], wherein they investigated passive and active eavesdropping attacks and proposed DL-based user scheduling schemes to enhance security, showing the flexibility and applicability of these techniques in GF-NOMA design.

Reconfigurable intelligent surfaces (RIS) have recently received attention owing to their ability to control wireless channels by changing impinging signals’ electromagnetic properties [39,40,41], wherein RIS control wireless channels to facilitate GF-NOMA by improving channel gain disparity among NOMA users and eliminate the need for power control, which is especially problematic for UL traffic. In [40], authors proposed a user pairing, RIS assignment, and phase shift alignment framework to utilize multiple RIS distributed across the cell area to realize a GF-NOMA network. In [41], authors optimally divided RIS into two partitions, each of which is aligned to one of the two NOMA users, and the size of each partition is adjusted to maximize the max–sum and max–min rate while satisfying UL and DL QoS requirements of bidirectional NOMA networks.

### 1.2. Paper Contributions and Novelty

This paper proposes synchronous GF-NOMA frameworks that substantially differ from the above works in that low-complexity power control and UE clustering are effectively integrated to facilitate the power-reception disparity required by the PD-NOMA. The main contributions of this paper can be summarized as follows:

- Two simple yet effective PD-NOMA schemes are proposed for the low-complexity power control required by the GF-NOMA concept. In single-level GF-NOMA (SGF-NOMA), all UEs are requested to transmit at the same power level through a broadcast RAR message. Even though all UEs transmit at the same power, the SGF-NOMA reaches power-reception disparity based on the channel gain disparity of UEs.
- To further improve SGF-NOMA, we propose a multi-level GF-NOMA (MGF-NOMA) approach that groups UEs into partitions based on sounding reference signals (SRS) signal strength. Then, partitions are assigned with different but identical power levels and shared with partition members through broadcast signals. The MGF-NOMA executes partitioning and power leveling dynamically depending on the available number of RBs and UEs awaiting admission. In addition to channel gain differences, the MGF-NOMA further improves the power-reception disparity by varying partitions’ power levels. Assuming channel reciprocity, the proposed schemes are also adopted to downlink (DL) power control for low-complexity operations.
- An iterative UE clustering scheme is proposed using the proposed low-complexity power control schemes as building blocks. The clustering algorithm iteratively pairs UEs awaiting admission with the available RBs to maximize the network sum or max–min rate. Therefore, the iterative clustering approach introduces extra power control while performing RB allocation. Finally, clustering information is shared with UEs by broadcasting RAR messages, and UEs can transmit on allocated RBs anytime without GA.

The MGF-NOMA is shown to provide a power control behavior very close to a GB benchmark with optimal power control and deliver 96–98% and 96–99.9% of the benchmark UL and DL max–sum rate. When the identical power level is set to match the same power consumption with other schemes, the SGF-NOMA is shown to deliver 87% and 88% UL max–sum rate of the MGF-NOMA and benchmark schemes, respectively. This clearly shows the efficacy of implicit power control imposed by the proposed UE clustering scheme, which only depends on readily available standard SRS signals and forms clusters in the order of milliseconds for a network with hundreds of UEs. From an energy-efficiency point of view, the SGF-NOMA is shown to deliver up to three orders of magnitude better max–sum rate performance, especially at transmission powers less than 5 dBm. Since the MGF-NOMA and optimal benchmarks enforce the strongest and weakest channel UEs to transmit at maximum and minimum transmit powers, respectively, the SGF-NOMA offers a significantly higher energy consumption fairness and network lifetime as all UEs consume equal transmit powers. If one also accounts for its low signal overhead and simplicity, the SGF-NOMA can be regarded as the best fit for low-power and low-complexity IoT devices. On the other hand, the MGF-NOMA is especially more suitable for DL transmission for three reasons: (1) the broadcasting of partitions’ power levels is redundant; (2) the BS exactly knows the active UEs, thus not affected by the MGF-NOMA’s dependence on the accurate estimation of active UEs to determine power levels; and (3) the MGF-NOMA’s relatively lower energy efficiency can be tolerated at the BS. Albeit its significant performance in max–sum rate, the MGF-NOMA turns an extremely low performance in max–min rate. This is mainly because max–min rate optimization requires all nodes to reach the same transmission rate, which does not comply with the leveled power approach. On the other hand, the SGF-NOMA delivers a much better max–min rate performance than the MGF-NOMA. Especially at low transmission power range up to −10 dBm, the SGF-NOMA yields $3\times 10^{6}$ MbpJ energy efficiency compared to $1\times 10^{7}$ MbpJ benchmark.

### 1.3. Notations and Paper Organization

Throughout the paper, sets and their cardinality are denoted with calligraphic and regular uppercase letters (e.g., |A|=A), respectively. Vectors and matrices are represented in lowercase and uppercase boldfaces (e.g., a and A), respectively. The *i*th member of a vector and set is denoted by a[i] and A{i}, respectively. Likewise, matrix A’s entry on *i*th row and *j*th column is denoted by A[i,j]. Subscripts *b*, *r*, and *u* are used for indexing the BS, RB/cluster, and UEs, respectively. The most frequent symbols and notations are summarized in Table 1 for readers’ convenience.

The remainder of the paper is organized as follows: Section 2 presents the considered network model. Section 3 discusses the problem formulation and explains the proposed solution methodology. Section 4 introduces the optimal benchmark and the proposed low-complexity power control schemes. Section 5 presents the algorithmic implementation of the clustering approaches. Lastly, Section 6 presents numerical results, and Section 7 concludes the paper by remarking on the key findings.

## 2. Network Model

We consider a cellular network consisting of a BS serving *U* UEs over *R* available RBs, each with *B* Hz bandwidth, whose index sets are denoted by U and R, respectively. Based on the underlying traffic characteristics, the BS controls the network and allocates resources to achieve various performance goals, e.g., max–sum rate, max–min fair rate, etc. The UE${}_{u}$ accesses the allocated RB with probability $\alpha_{u}\in \left[0,1\right]$, which also depends on traffic characteristics of its communication type, e.g., HTC, MTC. Through long-term observation of access requests, the BS is assumed to have an accurate estimate of $\alpha_{u}$. It is worth noting that exact information on active UEs in the DL direction is already available to the BS.

To demonstrate the massive connectivity capability of the proposed GF-NOMA schemes, we primarily focus on network scenarios satisfying U≫R such that an RB/cluster can admit up to $N=\left\lceil \frac{U}{R}\right\rceil$ UEs, which is referred to as the maximum cluster size. The set of UEs allocated to operate on RB${}_{r}$ is referred to as a cluster that is represented by set $C_{r}=\left\{u\;|\;\chi_{r}^{u}=1,\forall u\in U,\sum_{u\in U}\chi_{r}^{u}\leq N\right\}$, where the binary clustering variable $\chi_{r}^{u}=1$ if UE${}_{u}$ is allocated to RB${}_{r}$, $\chi_{r}^{u}=0$ otherwise, i.e., there is a one-to-one correspondence between RB${}_{r}$ and $r^{th}$ UE cluster, $C_{r}$, which are used interchangeably throughout the paper. Since cluster members randomly access the channel, the set of cluster members currently active at the RB${}_{r},r\in R,$ is given by ${\tilde{C}}_{r}=\left\{u\;|\;\beta_{u}=1,\forall u\in C_{r}\right\}$, ${\tilde{C}}_{r}\subseteq C_{r}$, where $\beta_{u}\in \left\{0,1\right\}$ is the binary indicator of channel access following from $\alpha_{u}$ and ${\tilde{C}}_{r}=\sum_{u\in {\tilde{C}}_{r}}\beta_{u}$.

**Figure 1:**
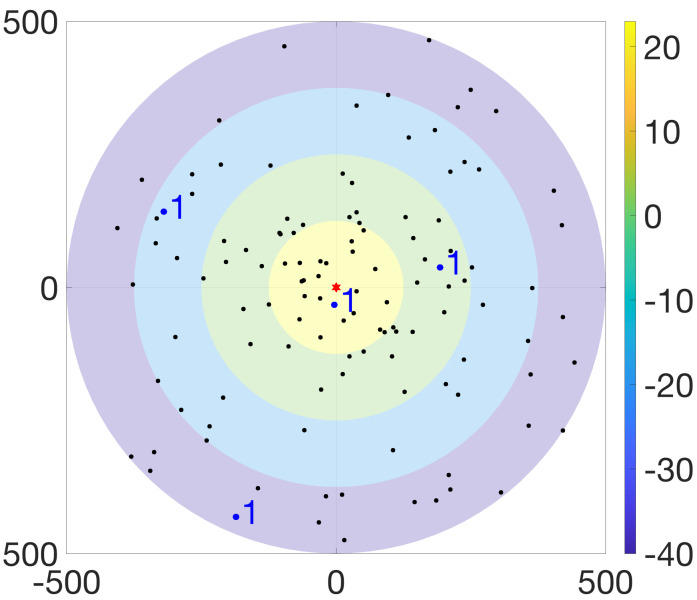
Illustration of UE partitioning for U=100, R=25, and N=4.

The BS and UEs operate in a single-input single-output (SISO) fashion, where the maximum transmission power of the BS is denoted by $P_{\max}^{b}$ that is shared equally across all RBs. Moreover, the maximum and minimum transmission powers of UEs are represented by $P_{\max}^{u}$ and $P_{\min}^{u}$, respectively. In the SGF-NOMA scheme, all UEs transmit at an identical power, denoted by $P_{\mathrm{id}}$. On the other hand, the MGF-NOMA divides the power control range $\left[P_{\min}^{u},P_{\max}^{u}\right]$ into *N* levels and requires UEs placed at level l∈{1,2,…,N} to adjust its transmission power to $P_{l}$. Figure 1 illustrates an aerial view of a network where the BS is located at the origin and serves 100 UEs over 25 RBs/clusters, each hosting 4 UEs. Although the rings represent four partitions, the ring colors indicate the transmission power of partitions such that UEs fall into yellow (level 1), green (level 2), blue (level 3), and purple (level 4) rings transmit at 23 dBm, 2 dBm, −19 dBm, and −40 dBm, respectively.

### Channel Model

All channels are assumed to be quasi-static, i.e., channel coherence time is longer than the time-slot duration during which UEs experience flat-fading. For a generic transmitter node *i* and receiver node *j*, the composite channel gain is given by

(1) $$h_{i}^{j}=10^{-\rho {}_{i}^{j}/10}g_{i}^{j},\;\left\{i,j\right\}\in \left\{b,u\right\},\;i\neq j,$$

where $g_{i}^{j}$ is the channel gain representing small-scale fading modeled by Rayleigh distribution and $\rho_{i}^{j}$ is the large-scale fading. Since man-made structures incur significant shadowing and scattering impact on the channel attenuation, the commonly exploited free-space path loss model is inadequate to capture real-life signal losses. Therefore, we consider the statistical features of the underlying urban environment, such as (i) the percentage of build-up area to the total land area, (ii) the number of buildings per unit area, and (iii) the statistical distribution of building heights. Accordingly, the spatial expectation of channel attenuation over the probabilities of having line-of-sight (LoS) and non-line-of-sight (NLoS) links is given by [42]

(2) $$\rho_{i}^{j}=\sum_{k\in \{\mathrm{LoS},\mathrm{NLoS}\}}\upsilon_{i}^{j}\left(k\right)\left({\mathrm{FSPL}}_{i}^{j}+\eta_{k}\right)\;\left[\mathrm{dB}\right],$$

where $\eta_{k},k\in \left\{\mathrm{LoS},\mathrm{NLoS}\right\},$ refers to the mean value of the excessive path loss over the free-space path loss (FSPL) between transceivers, which is expressed as

$${\mathrm{FSPL}}_{i}^{j}=20\left[\log_{10}\left(d_{i}^{j}\right)+\log_{10}\left(f_{c}\right)+\log_{10}\left(\frac{4\pi }{c}\right)\right]\left[\mathrm{dB}\right],$$

where $d_{i}^{j}$ is the distance between transceivers, $f_{c}$ is the carrier frequency, and *c* is the speed of light. Denoting the heights by $H_{i}$ and $H_{j}$, the probability of having an LoS transmission is given by

(3) $$\begin{array}{cc} \upsilon_{i}^{j}\left(\mathrm{LoS}\right) & \frac{1}{1+a_{i}\exp\left[-b_{i}\left(\arctan\left(\frac{H_{j}-H_{i}}{d_{i}^{j}}\right)-a_{i}\right)\right]}, \end{array}$$

where $a_{i}$ and $b_{i}$ are the approximation parameters depending on $H_{i}$/$H_{j}$, the mean number of buildings per km${}^{2}$, distribution of building heights, and the ratio of lands covered by buildings to the total land area [1]. The probability of having NLoS links directly follows from (3) as $\upsilon_{i}^{j}\left(\mathrm{LoS}\right)=1-\upsilon_{i}^{j}\left(\mathrm{NLoS}\right)$.

## 3. Problem Definition and Solution Methodology

In this section, we first provide joint power control and user clustering formulation of the optimal PD-NOMA scheme, then provide an overview of the proposed GF-NOMA frameworks.

### 3.1. Problem Definition

The DL sum rate maximization problem can be formulated as follows

(4) $$\;\begin{array}{cccc} \; & \;P_{s}:\max_{\begin{array}{c} \chi_{r}^{u},\omega_{r}^{u}\\ \forall r,\forall u \end{array}}\; & \; & \;\sum_{\forall u\in {\tilde{C}}_{r},\forall r\in R}\gamma_{r}^{u}\left(\omega_{r}\right)\chi_{r}^{u}\\ \; & \;s.t.\\ \; & \;C_{s}^{1}:\;\; & \; & \;\gamma_{r}^{u}\left(\omega_{r}\right)\chi_{r}^{u}\geq 2^{\lambda_{u}/B}-1,\;\forall u\in {\tilde{C}}_{r},\forall r\in R,\\ \; & \;C_{s}^{2}: & \; & \;\sum_{r\in R}\chi_{r}^{u}\leq 1,\;\forall u\in U,\\ \; & \;C_{s}^{3}: & \; & \;\sum_{\forall u\in U}\chi_{r}^{u}\leq N,\;\;\forall r\in R,\;//\;\mathrm{omitted}\;\mathrm{in}\;\mathrm{the}\;\mathrm{UL}\\ \; & \;C_{s}^{4}: & \; & \;\sum_{\forall u\in {\tilde{C}}_{r}}\omega_{r}^{u}\leq 1,\;\forall r\in R,\\ \; & \;C_{s}^{5}: & \; & \;\chi_{r}^{u}\in \left\{0,1\right\},\omega_{r}^{u}\in \left[0,1\right],\forall r,\forall u \end{array},$$

where $\omega_{r}$ is the power allocation vector, $C_{s}^{1}$ ensures that the SINR of each UE, $\gamma_{r}^{u}\left(\omega_{r}\right),$$\forall u\in {\tilde{C}}_{r}$, ∀r∈R, satisfy the QoS requirement $\lambda_{u}$ [bps], $C_{s}^{2}$ assures each UE is allocated at most one cluster, $C_{s}^{3}$ limits the cluster size by N=⌈U/R⌉, $C_{s}^{4}$ is the constraint on total cluster transmission power, and $C_{s}^{5}$ specifies the domain and bounds on optimization variables. The UL sum rate maximization problem can be formulated as in (4) by omitting the constraint on total cluster transmission power, $C_{s}^{4}$, as each UE has its own power source in the UL transmission. By introducing an auxiliary variable ψ and enforcing all UEs to reach an SINR no less than ψ, the DL max–min fair rate problem can be formulated as follows

(5) $$\;\begin{array}{cccc} \; & \;P_{f}:\max_{\begin{array}{c} \psi ,\chi_{r}^{u},\omega_{r}^{u}\\ \forall r,\forall u \end{array}}\; & \; & \;\psi \\ \; & \;s.t.\; & \; & \;\gamma_{r}^{u}\left(\omega_{r}\right)\chi_{r}^{u}\geq \psi ,\;\forall u\in {\tilde{C}}_{r},\forall r\in R,\\ \; & \; & \; & \;C_{s}^{2}-C_{s}^{5} \end{array},$$

which can be further reduced to the UL max–min fair rate problem by ignoring $C_{s}^{4}$ as in the max–sum rate problem. $P_{m}$ and $P_{f}$ are both mixed-integer non-linear programming (MINLP) problems, whose computational complexity is prohibitively high to employ in real life even for a moderate size of the network. Although the binary clustering variables cause the mixed-integer nature, the non-linearity results from power control variables. Given a certain clustering solution, the non-linear power control problem is indeed non-convex due to the interference terms in the SINR expressions, which are defined in the next section. Even though variations of these problems are studied extensively in the PD-NOMA literature, the underlying complexity of power control is not suitable to the spirit of GF-NOMA, as explained before. Therefore, we will benchmark the solution to these problems against the proposed low-complexity GF-NOMA frameworks presented next.

### 3.2. Solution Methodology

**Figure 2:**
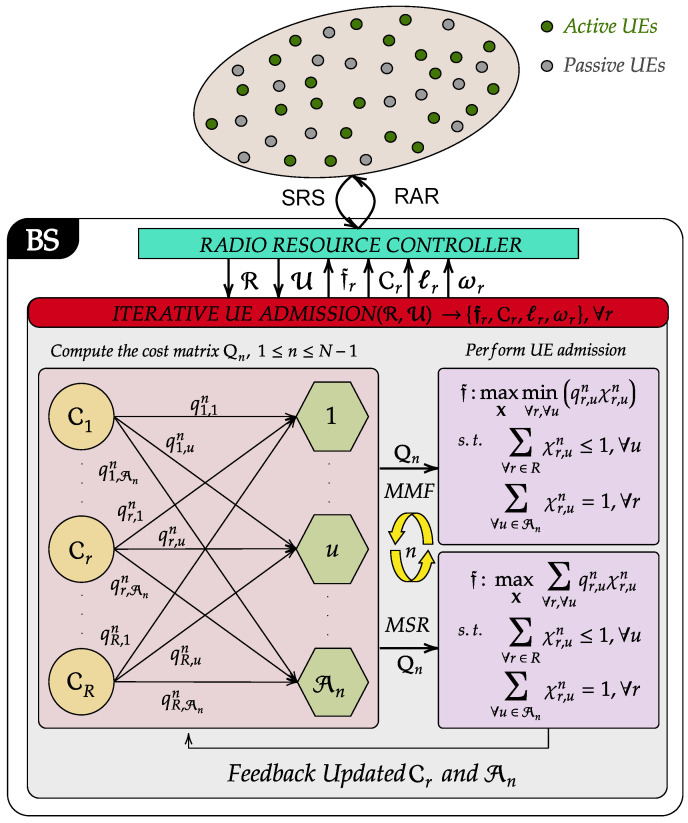
Schematic illustration of the solution methodology.

As shown in Figure 2, the proposed GF-NOMA framework operates merely on the received signal strength (RSS) of SRS, which is a Zadoff-Chu sequence transmitted by each UE separately from PUSCH and physical uplink control channel (PUCCH). UEs can transmit SRS on any subcarriers in the last symbol in an uplink subframe regardless of subcarriers assigned to another channel. For the sake of channel reciprocity, we focus on TDD mode, where SRS can also be sent in the last two symbols of the special subframe if the uplink pilot time slot (UpPTS) is configured to be long enough. Based on the RSS, the network controller computes the UL power levels L and DL power weights W to form a look-up table. Then, the proposed clustering approach iteratively forms clusters based on input parameters R, U, L, and W. The $n^{th},n\in \left[1,N-1\right],$ iteration starts with creating bi-partite matching weights, i.e., cost matrix entities, between RBs/clusters and the set of UEs awaiting admission $A_{n}$. After that, the cost matrix is used to perform UE admission by solving linear sum or linear bottleneck assignments for max–sum rate and max–min rate objectives, respectively. Following the user-cluster assignment, clusters are updated and prepared for the next phase of the UE admission. Once the admissions are finalized, the clustering algorithm returns the cluster sets $C_{r}$ along with their fitness $f_{r},\forall r$. Finally, allocated RBs and power levels are encapsulated into RAR messages and broadcast to UEs. Further details of the proposed scheme are provided in the next two sections.

## 4. A Low-Complexity PD-NOMA

In the DL (UL) NOMA schemes, the BS (UEs) perform successive interference cancellations, where the messages broadcast (transmitted) from the BS (UEs) are decoded in the descending order of signal reception power. It is worth noting that the optimal power allocation strategy is the opposite for UL and DL transmissions. The optimal UL-NOMA scheme ensures the strongest channel gain UE has the highest reception power at the BS such that remaining cluster members have more room to cause interference to improve their SINRs. This is typically achieved by forcing the UE with the strongest channel gain to transmit maximum power. On the contrary, the optimal DL-NOMA ensures the weakest channel gain UE has the highest reception power and experiences interference from the stronger users in the cluster. In this case, the strongest channel gain UE is allocated with the lowest power weight while enjoying substantially reduced interference after the SIC procedure. It is worth noting that a residual interference is possible after the SIC procedure due to hardware imperfections and inaccurate channel estimation [17,18]. Therefore, we numerically evaluate the impact of SIC imperfections in Section VII. In light of the above discussions, this section first formulates the optimal PD-NOMA benchmark. Then, it introduces the low-complexity power control developed explicitly for the proposed GF-NOMA approach.

### 4.1. Benchmark Optimal PD-NOMA

The benchmark scheme requires UEs’ exact CSI to calculate optimal transmission powers to reach the objective function of interest. For the $r^{th}$ cluster, ${\tilde{C}}_{r},\forall r$, we denote the power weight, composite channel gain, and received power vectors by $\omega_{r}$, $h_{r}$, and $p_{r}$, respectively. Assuming the reciprocity of DL and UL channels, the optimal received powers in DL and UL transmissions are given by $p_{r}=\frac{1}{R}P_{\max}^{b}\omega_{r}h_{r}$ and $p_{r}=P_{\max}\omega_{r}h_{r}$, respectively. Likewise, the optimal received powers sorted in descending order are represented by ${\overset{ˇ}{p}}_{r}$ with ${\overset{ˇ}{p}}_{r}\left[i\right]<{\overset{ˇ}{p}}_{r}\left[j\right],j>i,\left\{i,j\right\}\in \left[1,C_{r}\right]$. Accordingly, the signal-to-interference-plus-noise-ratio (SINR) of $i^{th}$ ordered member of $C_{r}$ is expressed as

(6) $${\tilde{\gamma }}_{r}^{i}\left(\omega_{r}\right)=\frac{{\overset{ˇ}{p}}_{r}\left[i\right]}{\sum_{\begin{array}{c} \forall j\in {\tilde{C}}_{r}\\ j>i \end{array}}{\overset{ˇ}{p}}_{r}\left[j\right]+\sigma^{2}},\forall i\in {\tilde{C}}_{r},$$

where $1⪰\omega_{r}⪰0$, $\sum_{\begin{array}{c} \forall j\in {\tilde{C}}_{r},\;j>i \end{array}}{\overset{ˇ}{p}}_{r}\left[j\right]$ is the intra-cluster interference, $\sigma^{2}=N_{0}B$ is the thermal noise power and $N_{0}$ is the noise power spectral density. When all cluster members are active, i.e., ${\tilde{C}}_{r}\equiv C_{r}$, Equation (6) is specially denoted by $\gamma_{r}^{u}$. The problem formulations for UL and DL optimal power control for max–min rate and max–sum rate objectives are presented in Table 2, where $\bar{\psi }=2^{\lambda /B}-1$. These can be reformulated as geometric programming problems as explained in [17,18,43,44] and optimal power control weight vectors ${\overset{★}{\omega }}_{r}$ can be obtained by using numerical solvers.

### 4.2. Proposed Low-Complexity PD-NOMA

In the MGF-NOMA, the BS orders UEs as per the RSS obtained from SRSs transmitted by UEs. The ordered UE sets are then partitioned into *N* distinct groups such that $U_{i}$ represents the ordered index set of *i*th highest RSS group of UEs with partition sizes $U_{i}=R,i\in \left\{1,2,\ldots ,N-1\right\},$ and $U_{N}=U\;\mathrm{mod}\;\left(R\right)$. The power control range of UEs is divided into *N* levels such that the dBm scaled maximum and minimum levels are given by $l_{\max}=10\log_{10}\left(P_{\max}^{u}\right)+30$ and $l_{\min}=10\log_{10}\left(P_{\min}^{u}\right)+30$, respectively. Accordingly, the linearly spaced transmit power level set is given by

(7) $$L\left(N\right)=\left\{l_{i}\left|l_{i}=l_{\max}-\frac{\left(i-1\right)\left(l_{\max}-l_{\min}\right)}{N-1},i\in \left[1,N\right]\right.\right\},$$

where $l_{i}$ [dBm] is the transmission power level of UEs belonging to $U_{i}$. By taking the union of transmit power levels for various cluster sizes, i.e., $L={⋃}_{k=1}^{K}L\left(k\right)$, the BS can form a look-up table, which is known by all UEs in advance. Based on the maximum and minimum UE output power specifications of LTE standards [45], Table 3 shows a power level look-up table for $l_{\max}=23$ dBm, $l_{\min}=-40$ dBm, and N≤10. Based on Table 3, the power level vector of the clusters, $l_{r},\forall r$, can be decided. For instance, in Figure 1, the highlighted cluster is allocated to RB${}_{r}$ with power level vector of highlighted cluster is $l_{r}=\left[1,10,20,30\right]$. Thus, the cluster members falling into yellow, green, blue, and purple rings receive RAR messages indicating (r,1), (r,10), (r,20), and (r,30); and transmit with power levels 23 dBm, 2 dBm, −19 dBm, and −40 dBm on RB${}_{r}$ freely until it receives another RAR, respectively. Accordingly, the SINR of $i^{th}$ ordered member of ${\tilde{C}}_{r}$ is expressed as

(8) $${\tilde{\gamma }}_{r}^{i}\left(l_{r}\right)=\frac{10^{(l_{r}\left[i\right]-30)/10}h_{r}\left[i\right]}{\sum_{\begin{array}{c} \forall j\in {\tilde{C}}_{r}\\ j>i \end{array}}10^{(l_{r}\left[j\right]-30)/10}h_{r}\left[j\right]+\sigma^{2}},\forall i\in {\tilde{C}}_{r},$$

where cluster members always transmit at a designated power level regardless of which members are active or passive. The second term of (8), $\sum_{\begin{array}{c} \forall j\in {\tilde{C}}_{r},\;j>i \end{array}}10^{(l_{r}\left[j\right]-30)/10}h_{r}\left[j\right]$, represents the intra-cluster interference. In the SGF-NOMA, the BS designates an identical power level and broadcasts an RAR message indicating the power level index to all UEs, which can be updated whenever necessary. Since the SGF-NOMA does not need to transmit individual power levels along with the RB indices for each UE, it incurs less signaling overhead than the MGF-NOMA.

Although the DL-NOMA schemes are not limited by signaling overhead, the computational complexity required to calculate the optimal power weights for the massive number of users is still prohibitive. Therefore, we further extend the proposed ultra low-complexity power control scheme to the DL-NOMA by translating the power level set in (7) into the DL power weights as follows

(9) $$W\left(N\right)=\left\{\omega_{i}\left|\omega_{i}=\frac{10^{(l_{i}-30)/10}}{\sum_{i}10^{(l_{i}-30)/10}},l_{i}\in \hat{L}\left(N\right)\right.\right\},$$

where $\hat{L}\left(N\right)$ is obtained by sorting L(N) in ascending order since the DL power weights follow opposite power weights as explained at the beginning of this section. Similar to the UL case, the BS can take the union of power weights for various cluster sizes to form a look-up table of weights $W={⋃}_{k=1}^{K}W\left(k\right)$. For instance, in Figure 1, the BS adjusts the power weight of UEs falling into yellow, green, blue, and purple rings to $\omega_{1}=4.97\times 10^{-7}$, $\omega_{2}=6.26\times 10^{-5}$, $\omega_{3}=7.9\times 10^{-3}$, and $\omega_{4}=0.9921$, respectively.

## 5. Iterative UE Admission

This section presents the iterative UE admission approach that integrates proposed power control schemes with RB allocation and UE clustering. Algorithm 1 provides the pseudo-code of the solution methodology pictorially depicted in Figure 2. For the sake of generalization, Algorithm 1 is presented for the MGF-NOMA framework, which can be simply reduced to the SGF-NOMA by considering a single partition and power level. In the remainder, we explain Algorithm 1 in more details:

Following the SRS reception from all UEs in Line 2, the BS determines the number of active UEs, maximum cluster size, and number of clusters in Line 3, Line 4, and Line 5, respectively. Due to the reverse operation characteristics of NOMA in UL and DL direction, Line 7 and Line 9 sort UEs in descending and ascending order of the channel quality for UL and DL iterative UE admission, respectively. Based on the obtained sorted UE index set I, Line 11 partitions the UE set into *N* subsets, i.e., $U_{n}=I\left\{\left(n-1\right)R+1:nR\right\},n\in \left[1,N\right]$. Then, the clusters are initialized in Line 12, where $C_{r}$ is initialized with the $r^{th}$ element of the first partition, ∀r.

The iterative UE admission is executed between Line 13 and Line 22 as follows: Line 14 updates the set of UEs awaiting admission. Then, Line 15 calls EvalCostMatrix procedure to compute the cost matrix of *n*th round of UE admission, $Q_{n}$, which is explained in the next paragraph. Then, Line 17 and Line 19 determine new admissions that maximizes the sumrate and minimum rate of all clusters by calling Linear Sum Assignment and Linear Bottleneck Assignment procedures, respectively. The assignment problems ensure that all clusters admit a UE if $R\leq A_{n}$ or all UEs are otherwise assigned to a cluster. There exist algorithms that can solve linear assignment problems in polynomial time. For example, the Jonker–Volgenant method has a cubic worst case complexity [46], i.e., $O\left({\left(K_{n}\right)}^{3}\right)$ where $K_{n}=\max\left\{R,A_{n}\right\}$. Burkard et al. proves in Theorem 6.4 in [47] that Linear Bottleneck Assignment can be solved in $O\left({\left(M_{n}\right)}^{2.5}\sqrt{\log M_{n}}\right)$, where $M_{n}=\min\left\{R,A_{n}\right\}$. They also developed a thresholding-based algorithm that uses Linear Sum Assignment and maximum cardinality bipartite matching Algorithm 6.1 in [47]. Based on assignment results, Line 21 updates the clusters for the next round of UE admission. Once the iterative user admission is finalized, power levels and weights of clusters are updated in Line 23 and Line 24, respectively. Finally, Line 25 sends RAR messages to UEs indicating allocated RB and assigned power levels.

The cost matrix $Q_{n}\in R^{R\times A_{n}}$ is computed by nested for loops between Line 28 and Line 49. At *j*th inner loop iteration, Line 30 forms a temporary cluster $T_{r}$ by admitting *j*th element of $A_{n}\left\{j\right\}$ into $C_{r}$. For the UL transmission, Line 32 determines the power levels as per the MGF-NOMA or SGF-NOMA schemes introduced in the previous section. Based on the objective function of interest, Line 34 and Line 36 compute $Q_{n}\left[i,j\right]$ by evaluating the UL max–sum and max–min rates of temporary cluster $T_{r}$, respectively. Similarly, Line 39 determines the DL power weights. Then, Line 41 and Line 43 compute $Q_{n}\left[i,j\right]$ by evaluating the DL max–sum and max–min rates of temporary cluster $T_{r}$, respectively. The final cost matrix is then returned for the UE assignment.
**Algorithm 1** Proposed MGF-NOMA and SGF-NOMA1:**Input:**R, O∈{MS,MM}, D∈{UL,DL}2:s←*Obtain SRS from UEs*3:U←*Determine active set of UEs*4:$N\leftarrow \left\lceil \frac{U}{R}\right\rceil$                          // *Determine maximum cluster size*5:C←R // *Determine number of clusters*6:**if** D=UL **then**7:    I←SortDescend($s_{u},\forall u$)                        // *UE ordering for UL*8:**else**9:    I←SortAscend($s_{u},\forall u$)                        // *UE ordering for DL*10:**end if**11:$U_{n}\leftarrow I\left\{\left(n-1\right)R+1:nR\right\},n\in \left[1,N\right]$                  // *Form UE partitions*12:$C_{r}\leftarrow U_{1}\left\{r\right\},\forall r$                              // *Initialize clusters*13:**for** n=1:N-1 **do**                          // *Iterative UE admission starts*14:    $A_{n}\leftarrow {⋃}_{i=n+1}^{N}U_{n}$                      // *Initialize admission awaiting UEs*15:    $Q_{n}\leftarrow$EvalCostMatrix($p,C_{r},A_{n}$)16:    **if** O=MS **then**                         // *Max–Sum Rate Assignment*17:        $f_{r},\chi \leftarrow$Linear Sum Assignment($Q_{n}$)18:    **else if** O=MM **then**                        // *Max–Min Rate Assignment*19:        $f_{r},\chi \leftarrow$Linear Bottleneck Assignment($Q_{n}$)20:    **end if**21:    $C_{r}\leftarrow C_{r}⋃A_{n}\left\{j\right\},\chi \left[r,j\right]=1,\forall r,\forall j$                    // *Update clusters*22:**end for**23:$l_{r}\leftarrow L\left(C_{r}+1\right),\forall r$                          // *Update cluster levels*24:$\omega_{r}\leftarrow W\left(C_{r}+1\right),\forall r$                         // *Update cluster weights*25:UE${}_{j}\leftarrow \left(r,l_{r}\left[j\right]\right),{\mathrm{UE}}_{j}\in C_{r},\forall j,\forall r$                      // *Send RARs out*26:**return** $f_{r}$, $C_{r}$, $l_{r}$, $\omega_{r}$27:**procedure** EvalCostMatrix($p,C_{r},A_{n}$)28:    **for** i=1:R **do**29:        **for** j=1:$A_{n}$ **do**30:           $T_{r}\leftarrow C_{r}⋃A_{n}\left\{j\right\}$                       // *Admit $j^{th}$ UE of $A_{n}$ to $C_{r}$*31:           **if** D=UL **then**32:               l←L(n+1)                        // *power levels as per* (7)33:               **if** O=MS **then**34:                   $Q_{n}\left[i,j\right]\leftarrow \sum_{k\in T_{r}}B\log_{2}\left(1+\gamma_{r}^{k}\left(l\right)\right)$35:               **else if** O=MM **then**36:                   $Q_{n}\left[i,j\right]\leftarrow \min_{k\in T_{r}}\left\{B\log_{2}\left(1+\gamma_{r}^{k}\left(l\right)\right)\right\}$37:               **end if**38:           **else if** D=DL **then**39:               ω←W(n+1)                       // *power weights as per* (9)40:               **if** O=MS **then**41:                   $Q_{n}\left[i,j\right]\leftarrow \sum_{k\in T_{r}}B\log_{2}\left(1+\gamma_{r}^{k}\left(\omega \right)\right)$42:               **else if** O=MM **then**43:                   $Q_{n}\left[i,j\right]\leftarrow \min_{k\in T_{r}}\left\{B\log_{2}\left(1+\gamma_{r}^{k}\left(\omega \right)\right)\right\}$44:               **end if**45:           **end if**46:        **end for**47:    **end for**48:**return** $Q_{n}$49:**end procedure**50:**procedure** Linear Sum Assignment($Q_{n}$)51:    $\overset{}{\chi }\leftarrow \max_{\chi }\;\;\sum_{\forall i,\forall j}Q_{n}\left[i,j\right]\chi \left[i,j\right]$52:           $s.t.\;\sum_{i}\chi \left[i,j\right]\leq 1,\sum_{j}\chi \left[i,j\right]=1$53:**return** $\overset{}{\chi }$54:**end procedure**55:**procedure**Linear Bottleneck Assignment($Q_{n}$)56:    $\overset{}{\chi }\leftarrow \max_{\chi }\left\{\min_{\forall i,\forall j}\left\{Q_{n}\left[i,j\right]\chi \left[i,j\right]\right\}\right\}$57:           $s.t.\;\sum_{i}\chi \left[i,j\right]\leq 1,\sum_{j}\chi \left[i,j\right]=1$58:**return** $\overset{}{\chi }$59:**end procedure**

For the max–sum rate objective, the overall time complexity of iterative user admission is given by

$$\sum_{n=1}^{N-1}O\left(RA_{n}+{\left(K_{n}\right)}^{3}\right)\overset{R\gg 1,A_{n}\gg 1}{\to }\sum_{n=1}^{N-1}O\left({\left(K_{n}\right)}^{3}\right),$$

where the first and second terms of the left-hand side are the complexity of cost matrix formation and Linear Sum Assignment. Since $K_{n}\gg 1$ in practice, the overall complexity can be approximated by the dominant term, which has cubic time complexity. Likewise, the overall time complexity for the max–min rate objective is given by

$$\sum_{n=1}^{N-1}O\left({\left(M_{n}\right)}^{2.5}\sqrt{\log M_{n}}\right),$$

where the complexity of cost matrix formation is omitted following the same reason above.

## 6. Numerical Results

Without loss of generality, we assume that UEs are uniformly distributed over a macrocell area of a radius of 500 m. Unless explicitly stated otherwise, the numerical results are obtained by Matlab using the default parameters summarized in Table 4. The number of available RBs is intentionally selected as R=25 to show the proposed GF-NOMA schemes’ capability of supporting mMTC, where many devices share an RB. For benchmarking, we compare the proposed GF-NOMA frameworks with an optimal scheme that exploits CVX’s geometric programming toolbox to solve problems formulated in Table 2. Obtained UL and DL optimal power control weights are then used in the cost matrix formation procedure of Algorithm 1. In the rest of this section, all presented results are obtained by averaging over 1000 network instances. The proposed GF-NOMA frameworks are compared with the optimal benchmark throughout the section. All results are presented in double y-axes plots, where the left and right y-axes with blue and red colors show the max–sum and max–min rates obtained by employing Linear Sum Assignment and Linear Bottleneck Assignment, respectively.

### 6.1. Benchmark Comparison

**Figure 3:**
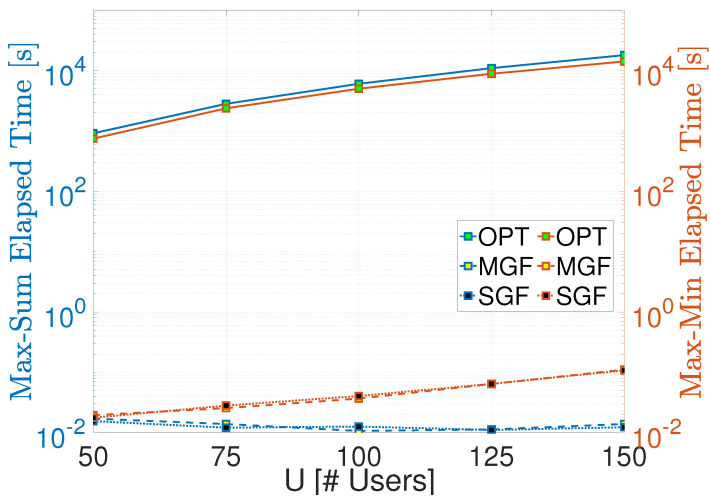
Time complexity compari1son.

Let us start with the elapsed time comparison of the proposed MGF-NOMA and SGF-NOMA frameworks. Figure 3 shows that the proposed frameworks have similar elapsed time performance, which increases with maximum cluster size $N=\lceil \frac{U}{R}\rceil$ such that U={50,75,…,150} corresponds to N={2,3,…,6}. Although the Linear Sum Assignment takes around 10 ms to finish the entire clustering, Linear Bottleneck Assignment ramps up to 100 ms, which is mainly due to the fact that it calls Linear Sum Assignment and maximum cardinality bipartite matching as sub-procedures. On the other hand, the optimal benchmark takes half an hour to three hours to complete, where the main complexity is due to the cost matrix formation, each element of which is computed using CVX.

**Figure 4:**
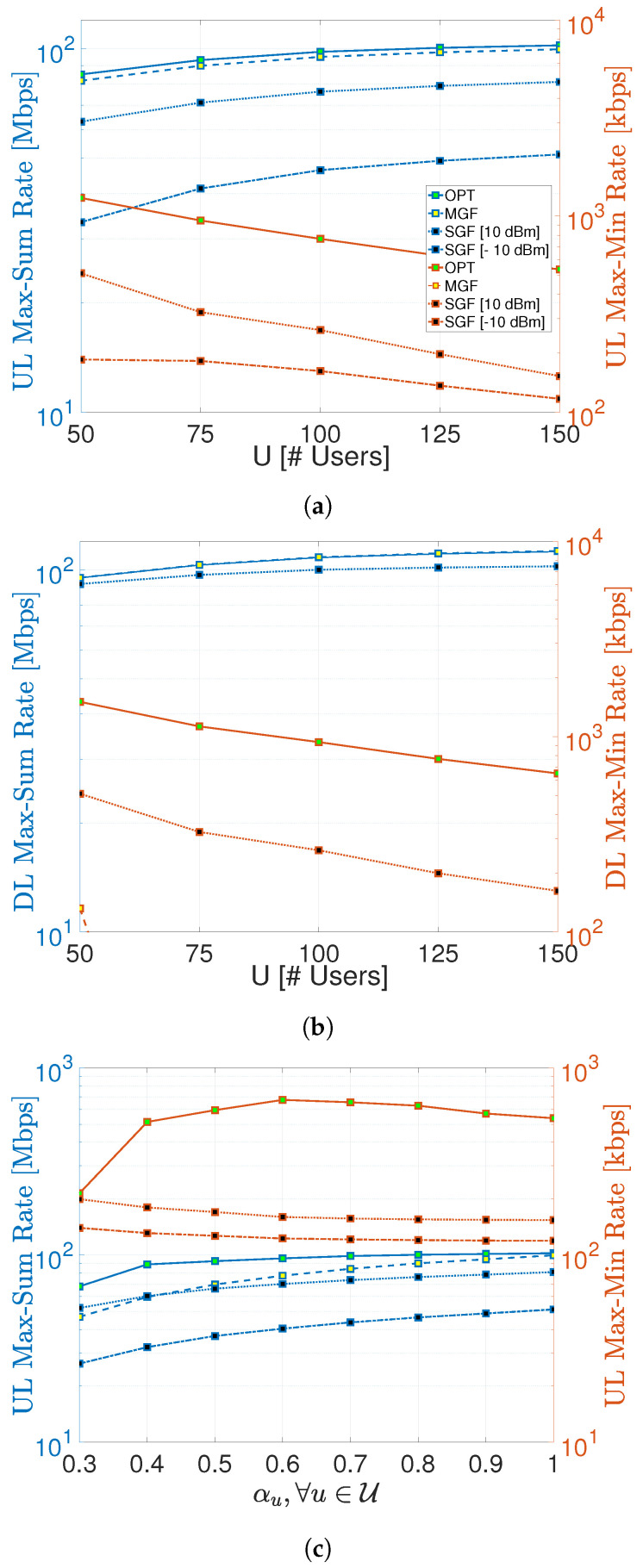
UL and DL max–sum rate and max–min rate comparison of proposed schemes. (**a**) UL max–sum rate and max–min rate. [$\alpha_{u}=1,\forall u\in U$]. (**b**) DL max–sum rate and max–min rate. [$\alpha_{u}=1,\forall u\in U$]. (**c**) UL max–sum rate and max–min rate. [U=150].

The max–sum and max–min rates of proposed GF-NOMA schemes are benchmarked against the optimal NOMA scheme in Figure 4, where left and right y-axes are scaled to Mbps and kbps for the sake of better visibility. Figure 4a shows that the MGF-NOMA scheme can reach 96–98% of the UL optimal max–sum rate. It is important to note that this slight difference is mainly caused by the power consumption difference, as explained in the next section. On the other hand, the SGF-NOMA scheme can reach 40–50% and 75–80% of the optimal UL max–sum rate for $P_{id}=-10$ dBm and $P_{id}=10$ dBm, respectively. Therefore, $P_{id}$ significantly impacts the SGF-NOMA performance, as the next section shows that the SGF-NOMA can reach above 90 % of the optimal UL max–sum rate under the same power consumption.

Although the MGF-NOMA provides a max–sum rate performance very close to the optimal benchmark, it delivers extremely poor max–min rate performance, which is 20 kbps at U=50 and sharply reduces to 0.25 kbps for U≥75. Such a huge performance degradation is due to the fact that leveled power control does not comply with the max–min rate power control’s main goal of having all nodes reach the same transmission rate. On the other hand, the SGF-NOMA can reach 15–22% and 29–41% of the optimal UL max–min rate for $P_{id}=-10$ dBm and $P_{id}=10$ dBm, respectively. Similar to the case in the max–sum rate, the $P_{id}$ substantially impacts the max–min rate performance, which is investigated more in detail in the next section.

Likewise, Figure 4b compares the DL max–sum and max–min rates of the proposed GF-NOMA frameworks with the optimal benchmark. Figure 4b shows that the MGF-NOMA and SGF-NOMA frameworks can reach 96–99.9% and 96–91% of the DL optimal max–sum rate, respectively. The drop from 96% to 91% is due to the fact that the SGF-NOMA equally shares the available DL transmission power among UEs, and it reduces as *U* increases. However, the max–min rate performance of the SGF-NOMA is not as good as the max–sum rate; it reaches 25–33% of the optimal max–min rate. Similar to the UL scenario, the MGF-NOMA also delivers the worst max–min rate performance, which starts with 132 kbps at U=50 and sharply reduces to 212 kbps for U≥75.

A common trend in Figure 4a,b is that the max–sum rate improves as *U* increases, mainly because an RB is more efficiently utilized as cluster size increases. On the contrary, the max–min rate degrades as cluster size increases since admitting more UEs into the same RB has a detrimental impact on the UEs performing worst. Notice that power control cannot simply eliminate this behavior, which all schemes share, including optimal benchmark.

Finally, Figure 4c shows the impact of the probability of being active on max–sum and max–min rates. It is worth reminding that only the MGF-NOMA scheme requires the number of active UEs to determine the proper power levels for UE partitions. The MGF curve in Figure 4c shows the performance at various UE activity scenarios when power levels are set, assuming all UEs are active. Therefore, the MGF curve converges to the optimal benchmark as α reaches unity. At this point, it is obvious that having a coarse estimate of *U* with ±R accuracy can still turn into a desirable performance compared to the optimal benchmark.

### 6.2. Date Rate, Power Consumption, and Energy-Efficiency Comparison

**Figure 5:**
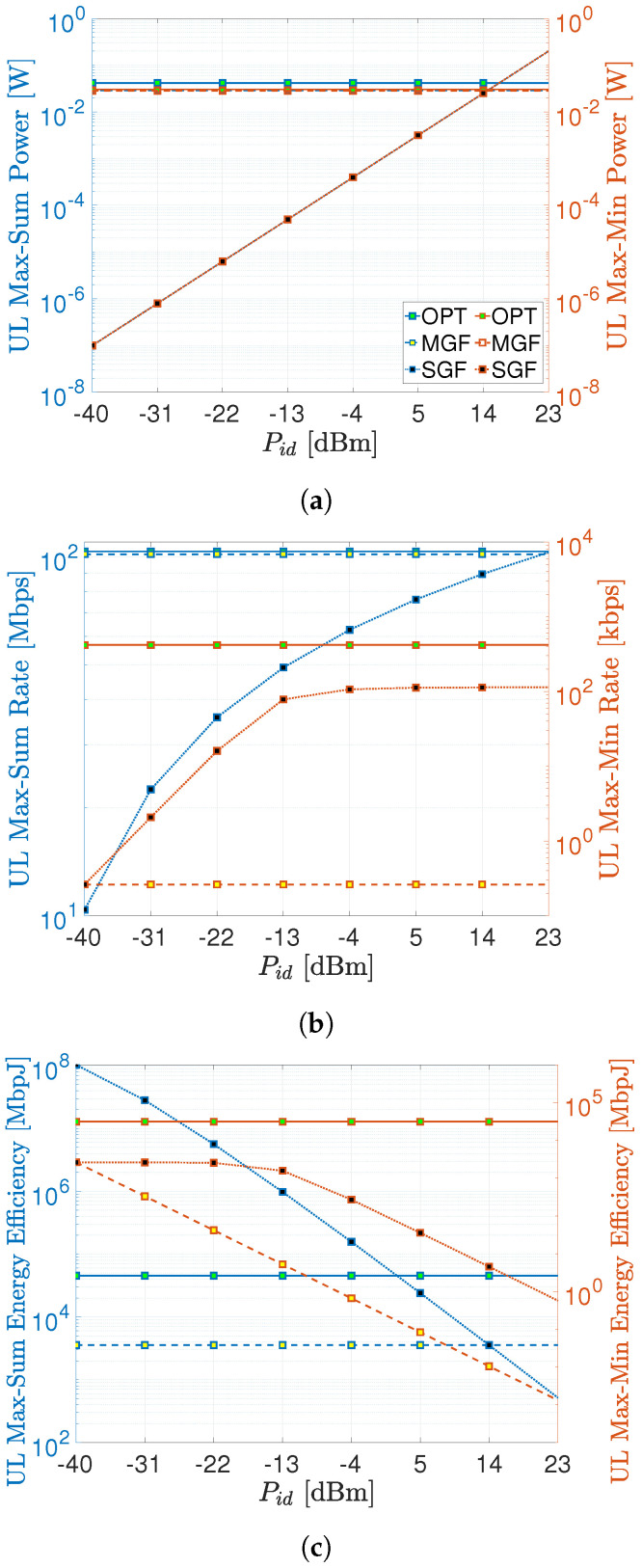
Data rate, power consumption, and energy-efficiency comparisons. [$U=150,\alpha_{u}=1,$∀u∈U]. (**a**) Average power consumption comparisons. (**b**) Max–sum rate and max–min rate comparisons. (**c**) Energy-efficiency comparisons.

As mentioned above, the $P_{id}$ substantially impacts the performance of SGF-NOMA, which is investigated more in depth in this section. Figure 5a shows the average power consumption per UEs with respect to $P_{id}$. The optimal benchmark consumes 50% more power than the MGF-NOMA to deliver around 2% higher max–sum rate as depicted in Figure 5b. On the other hand, the optimal max–min rate benchmark consumes 27.5% less than the optimal max–sum rate benchmark. It is obvious from Figure 5a that the SGF-NOMA consumes the same power consumption as the MGF-NOMA and optimal benchmark when $P_{id}$ reaches 15 and 16.5 dBm, respectively. At the same power consumption levels, the SGF-NOMA can reach 87% and 88% of the optimal benchmark and MGF-NOMA max–sum rate, respectively. On the other hand, the SGF-NOMA can reach a 25% max–min rate of the optimal benchmark starting from $P_{id}=-13$ dBm, which is around 100 times less power consumption than the optimal benchmark. However, increasing $P_{id}$ does not improve the max–min rate beyond $P_{id}=-13$ dBm.

At this point, it is crucial to compare these approaches from an energy-efficiency point of view since most IoT nodes are designed as low-power devices to increase their cost and lifetime [48]. As shown in Figure 5c, the optimal benchmark always has better energy efficiency than the MGF-NOMA, i.e., the aforementioned 2% max–sum rate enhancement in return for 50% more consumption yields a significant energy-efficiency improvement for the optimal benchmark. Even though the SGF-NOMA delivers a lower max–sum rate up to around $P_{id}=0$ dBm, Figure 5c shows it has the highest energy efficiency in this region. For the max–min rate case, the optimal benchmark, and the MGF-NOMA always deliver the highest and the lowest energy-efficiency performance, respectively. The SGF-NOMA turns relatively better energy efficiency, which constantly reduces after $P_{id}=-13$ dBm. Finally, we summarize the best value of $P_{id}$ for various performance metrics under max–sum rate and max–min rate operational regimes in Table 5.

### 6.3. Impact of SIC Imperfections on System Performance

In real life, a perfect SIC operation is not always possible due to the imperfections caused by CSI acquisition errors and hardware impairments.

- **Decoding Errors:** The success of SIC largely depends on the accurate decoding of the strongest signal. If there is an error in the decoding of the strongest user, this error becomes propagated when subtracting it from the combined signal, affecting the decoding of the next user.
- **Receiver Non-linearities:** Even with perfect decoding, there might be residual interference after subtraction due to non-linearities in the receiver, which can degrade the performance of the subsequent user’s signal decoding.
- **Channel Estimation Errors:** For the subtraction to be perfect, the receiver must accurately estimate the channel conditions. Any error in channel estimation will lead to imperfect cancellation.
- **Out-of-Order Decoding:** The assumption that users can be perfectly ordered according to their channel conditions may not always hold, especially in dynamic environments. Decoding a user out of order can degrade the performance of SIC.

**Figure 6:**
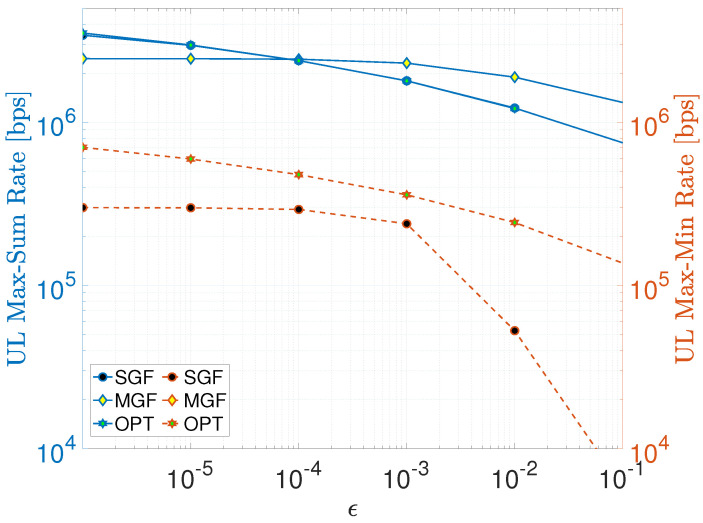
Impact on residual SIC interference.

One way of showing detrimental impacts of imperfect SIC operation is quantifying the residual error after each SIC operation using an SIC error factor 0≤ϵ≤1 [17,18,43,44], which can be incorporated into the SINR expression in (10) as follows

(10) $${\tilde{\gamma }}_{r}^{i}\left(\omega_{r}\right)=\frac{{\overset{ˇ}{p}}_{r}\left[i\right]}{\epsilon \sum_{\begin{array}{c} \forall j\in {\tilde{C}}_{r}\\ j<i \end{array}}{\overset{ˇ}{p}}_{r}\left[j\right]+\sum_{\begin{array}{c} \forall j\in {\tilde{C}}_{r}\\ j>i \end{array}}{\overset{ˇ}{p}}_{r}\left[j\right]+\sigma^{2}},\forall i\in {\tilde{C}}_{r},$$

where the first term in the denominator represents the total residual interference after SIC due to the aforementioned imperfections, whereas the second term is non-cancellable intra-cluster interference. To investigate the impact of residual interference on max–sum and max–min rates, let us content ourselves with the UL scenario and set the identical transmit power of SGF-NOMA to the mean power consumption of MGF-NOMA (i.e., $P_{id}\approx 16$ dBm) for a fair comparison. Figure 6 compares the impact of increasing the SIC error factor on SGF-NOMA, MGF-NOMA, and OPT-NOMA schemes on the average cluster sum and max–min rate. For the max–sum rate, SGF-NOMA provided a performance close to OPT-NOMA that exploits the CVX solver to optimize power levels by taking ϵ into account. Even if the proposed schemes are not designed to account for SIC imperfection factor ϵ, their performance is noteworthy as they deliver a performance comparable to the optimal scheme that adjusts power weights to mitigate the impact of residual interference. This was possible due to the implicit power control through the proposed clustering approach. On the other hand, MGF-NOMA provides better immunity against SIC imperfections starting from $\epsilon =10^{-4}$. Nonetheless, all schemes suffer from increasing residual interference regardless of underlying the power control and clustering approach. The influence of optimal power control is more significant in the case of max–min fairness, where OPT-NOMA outperforms SGF-NOMA, especially for $\epsilon \geq 10^{-3}$. Notice that MGF-NOMA is not shown in Figure 6 as it delivers around 50 bps max–min rate performance, which is much less than $10^{4}$ bps.

**Table 5:** The best value of $P_{id}$ for various performance metric under different regimes.

	Performance Metrics		
**Operational Regime**	**Power Consumption**	**Data Rate**	**Energy Efficiency**
**Max–Sum Rate**	14.50 [dBm]	23.00 [dBm]	−40.00 [dBm]
**Max–Min Rate**	15.25 [dBm]	23.0 [dBm]	2.00 [dBm]

## 7. Conclusions and Future Research Directions

This paper introduces a synchronous GF-NOMA framework that seamlessly integrates straightforward yet highly efficient power control techniques with UE clustering and RB allocation strategies. The results obtained from these methods demonstrate the remarkable capability to form clusters within milliseconds and achieve max–sum rates that closely approach the optimal benchmark. However, it is worth noting that the proposed frameworks exhibit relatively lower performance when compared to the optimal benchmark in terms of max–min fairness. Therefore, future research endeavors should be directed towards the development of low-complexity power control schemes aimed at further enhancing the max–min rate performance.

In addition, an intriguing avenue for exploration lies in extending the proposed frameworks to MIMO systems. Hybridizing spatial NOMA schemes with the proposed approach in MIMO configurations holds the potential to yield fascinating insights and performance improvements. Furthermore, there is considerable promise in leveraging deep-learning methodologies for tasks such as user activity detection and dynamic power level determination based on underlying network parameters. These machine-learning techniques can potentially enhance the adaptability and optimization of GF-NOMA systems.

Another vital area ripe for future research is the security aspects of GF-NOMA. This includes a comprehensive exploration of vulnerability analysis, the development of robust encryption techniques, and the creation of innovative methods to safeguard against eavesdropping attacks, which become particularly challenging in grant-free operations where traditional user authentication mechanisms through grant acquisition are absent.

## Figures and Tables

**Table 1:** Table of Notations.

Nots.	Description
U	Set of *U* users
R	Set of *R* RBs
*N*	Cluster size, N=⌈U/R⌉
*B*	RB bandwidth
$\alpha_{u}$	Access probability of UE${}_{u},u\in U$
$\chi_{r}^{u}$	Clustering binary indicator $\chi_{r}^{u}=1$ if UE${}_{u}$ is assigned to RB${}_{r}$, 0 otherwise
$C_{r}$	Cluster set of $C_{r}$ users belonging to RB${}_{r},\forall r\in R$
${\tilde{C}}_{r}$	Set of active members of $C_{r},r\in R$
$P_{\max}^{u}$	Maximum transmit power of UE${}_{u}\in U$
$P_{\min}^{u}$	Minimum transmit power of UE${}_{u}\in U$
$h_{i}^{j}$	Composite channel gain between generic nodes *i* and *j*
$\gamma_{r}^{u}$	SINR of UE${}_{u}$ at RB${}_{r}$
$\lambda_{u}$	QoS requirement of UE${}_{u}\in U$
$\omega_{r}$	Power allocation vector for $C_{r}$
L(N)	Linearly spaced *N* power transmit levels, $l_{1},\ldots ,l_{N}$
W(N)	*N* levels of DL transmit power, $\omega \_1,\ldots ,\omega_{N}$
$A_{n}$	Number of users awaiting cluster admission
$Q_{n}$	$R\times A_{n}$ cost matrix of $n^{th}$ user admission iteration

**Table 2:** Problem formulations for optimal max–min fair and max–sum rate PD-NOMA schemes.

${\overset{★}{\omega }}_{r}$	Uplink PD-NOMA	Downlink PD-NOMA
**Max–Min**	$\max_{\omega_{r},\psi }\;\psi \;\left(s.t.\right)\;{\tilde{\gamma }}_{r,i}\left(\omega_{r}\right)\geq \psi ,\;\forall i\in {\tilde{C}}_{r}$	$\max_{\omega_{r},\psi }\;\psi \;\left(s.t.\right)\;{\tilde{\gamma }}_{r,i}\left(\omega_{r}\right)\geq \psi ,\;\sum_{i}\omega_{i}\leq 1,\forall i\in {\tilde{C}}_{r}$
**Max–Sum**	$\max_{\omega_{r}}\;\sum_{i}{\tilde{\gamma }}_{r,i}\left(\omega_{r}\right)\;\left(s.t.\right)\;{\tilde{\gamma }}_{r,i}\left(\omega_{r}\right)\geq \bar{\psi },\;\forall i\in {\tilde{C}}_{r}$	$\max_{\omega_{r}}\;\sum_{i}{\tilde{\gamma }}_{r,i}\left(\omega_{r}\right)\;\left(s.t.\right)\;{\tilde{\gamma }}_{r,i}\left(\omega_{r}\right)\geq \bar{\psi },\;\sum_{i}\omega_{i}\leq 1,\forall i\in {\tilde{C}}_{r}$

**Table 3:** Power level look-up table for $P_{\min}^{u}=-40$ dBm, $P_{\max}^{u}=23$ dBm, and N≤10.

Level Indices	Powers [dBl	N (Cluster Size)								
		**2**	**3**	**4**	**5**	**6**	**7**	**8**	**9**	**10**
** $l_{1}$ **	23	✓	✓	✓	✓	✓	✓	✓	✓	✓
** $l_{2}$ **	16	–	–	–	–	–	–	–	–	✓
** $l_{3}$ **	15.125	–	–	–	–	–	–	–	✓	–
** $l_{4}$ **	14	–	–	–	–	–	–	✓	–	–
** $l_{5}$ **	12.5	–	–	–	–	–	✓	–	–	–
** $l_{6}$ **	10.4	–	–	–	–	✓	–	–	–	–
** $l_{7}$ **	9	–	–	–	–	–	–	–	–	✓
** $l_{8}$ **	7.25	–	–	–	✓	–	–	–	✓	–
** $l_{9}$ **	5	–	–	–	–	–	–	✓	–	–
** $l_{10}$ **	2	–	–	✓	–	–	✓	–	–	✓
** $l_{11}$ **	−0.625	–	–	–	–	–	–	–	✓	–
** $l_{12}$ **	−2.2	–	–	–	–	✓	–	–	–	–
** $l_{13}$ **	−4	–	–	–	–	–	–	✓	–	–
** $l_{14}$ **	−5	–	–	–	–	–	–	–	–	✓
** $l_{15}$ **	−8.5	–	✓	–	✓	–	✓	–	✓	–
** $l_{16}$ **	−12	–	–	–	–	–	–	–	–	✓
** $l_{17}$ **	−13	–	–	–	–	–	–	✓	–	–
** $l_{18}$ **	−14.8	–	–	–	–	✓	–	–	–	–
** $l_{19}$ **	−16.375	–	–	–	–	–	–	–	✓	–
** $l_{20}$ **	−19	–	–	✓	–	–	✓	–	–	✓
** $l_{21}$ **	−21	✓	–	–	–	–	–	–	–	–
** $l_{22}$ **	−22	–	–	–	–	–	–	✓	–	–
** $l_{23}$ **	−24.25	–	–	–	✓	–	–	–	✓	–
** $l_{24}$ **	−26	–	–	–	–	–	–	–	–	✓
** $l_{25}$ **	−27.4	–	–	–	–	✓	–	–	–	–
** $l_{26}$ **	−29.5	–	–	–	–	–	✓	–	–	–
** $l_{27}$ **	−31	–	–	–	–	–	–	✓	–	–
** $l_{28}$ **	−32.125	–	–	–	–	–	–	–	✓	–
** $l_{29}$ **	−33	–	–	–	–	–	–	–	–	✓
** $l_{30}$ **	−40	–	✓	✓	✓	✓	✓	✓	✓	✓

**Table 4:** Table of default parameters [1,45].

Param.	Value	Param.	Value	Param.	Value
*U*	150	$P_{\max}^{u}$	23 dBm	$\eta_{LoS}$	1.6
*R*	25	$P_{\min}^{u}$	−40 dBm	$\eta_{NLoS}$	20
*B*	180 kHz	$P_{\max}^{b}$	46 dBm	*a*	8
$N_{0}$	−174 dBm	$f_{c}$	1.8 GHz	*b*	0.2
λ	0.1 Mbps	$H_{BS}$	50 m	$H_{UE}$	2 m

## Data Availability

Not applicable.
